# TIEG1/KLF10 Modulates Runx2 Expression and Activity in Osteoblasts

**DOI:** 10.1371/journal.pone.0019429

**Published:** 2011-04-29

**Authors:** John R. Hawse, Muzaffer Cicek, Sarah B. Grygo, Elizabeth S. Bruinsma, Nalini M. Rajamannan, Andre J. van Wijnen, Jane B. Lian, Gary S. Stein, Merry Jo Oursler, Malayannan Subramaniam, Thomas C. Spelsberg

**Affiliations:** 1 Department of Biochemistry and Molecular Biology, Mayo Clinic, Rochester, Minnesota, United States of America; 2 Division of Cardiology, Department of Medicine, Northwestern University Feinberg School of Medicine, Chicago, Illinois, United States of America; 3 Department of Cell Biology and Cancer Center, University of Massachusetts Medical School, Worcester, Massachusetts, United States of America; 4 Endocrine Research Unit, Mayo Clinic, Rochester, Minnesota, United States of America; Texas A&M University, United States of America

## Abstract

Deletion of TIEG1/KLF10 in mice results in a gender specific osteopenic skeletal phenotype with significant defects in both cortical and trabecular bone, which are observed only in female animals. Calvarial osteoblasts isolated from TIEG1 knockout (KO) mice display reduced expression levels of multiple bone related genes, including Runx2, and exhibit significant delays in their mineralization rates relative to wildtype controls. These data suggest that TIEG1 plays an important role in regulating Runx2 expression in bone and that decreased Runx2 expression in TIEG1 KO mice is in part responsible for the observed osteopenic phenotype. In this manuscript, data is presented demonstrating that over-expression of TIEG1 results in increased expression of Runx2 while repression of TIEG1 results in suppression of Runx2. Transient transfection and chromatin immunoprecipitation assays reveal that TIEG1 directly binds to and activates the Runx2 promoter. The zinc finger containing domain of TIEG1 is necessary for this regulation supporting that activation occurs through direct DNA binding. A role for the ubiquitin/proteasome pathway in fine tuning the regulation of Runx2 expression by TIEG1 is also implicated in this study. Additionally, the regulation of Runx2 expression by cytokines such as TGFβ1 and BMP2 is shown to be inhibited in the absence of TIEG1. Co-immunoprecipitation and co-localization assays indicate that TIEG1 protein associates with Runx2 protein resulting in co-activation of Runx2 transcriptional activity. Lastly, Runx2 adenoviral infection of TIEG1 KO calvarial osteoblasts leads to increased expression of Runx2 and enhancement of their ability to differentiate and mineralize in culture. Taken together, these data implicate an important role for TIEG1 in regulating the expression and activity of Runx2 in osteoblasts and suggest that decreased expression of Runx2 in TIEG1 KO mice contributes to the observed osteopenic bone phenotype.

## Introduction

The TGFβ inducible early gene-1 (TIEG1), also known as KLF10, is a member of the Krüppel family of transcription factors which was originally identified as a primary response gene following TGFβ1 treatment of human osteoblasts [1]. Expression of TIEG1 is also rapidly induced by estrogen [2], BMPs [3], and EGF [1]. TIEG1 encodes a 480 amino acid protein which contains several Src homology domains in its N-terminal region and three C_2_H_2_ type zinc finger DNA binding domains in its C-terminal region [4]. Previously, we demonstrated that TIEG1 potentiates TGFβ1/Smad signaling through repression of the inhibitory Smad7 gene and activation of the Smad2 gene [5], [6]. Over-expression of TIEG1 mimics the effects of TGFβ1 in a number of cell types including osteoblasts [7] and pancreatic carcinoma cells [8]. Expression of TIEG1 in cancer cells is known to inhibit cell proliferation [7] and induce apoptosis [8], [9]. Moreover, TIEG1 levels inversely correlate with the progression and stages of breast cancer [10].

In addition to the inhibition of cell proliferation and induction of apoptosis, many other biological functions of TIEG1 have been identified through the development and characterization of knockout (KO) mice [11] including essential roles for this gene in normal heart [12], tendon [13], [14] and immune system [15], [16] function. Our laboratory has also verified a role for TIEG1 in regulating skeletal development and maintenance. TIEG1 KO mice exhibit a gender-specific osteopenic phenotype characterized by decreased bone mineral density, bone mineral content and overall loss of bone strength only in female animals [17], [18]. At the cellular level, calvarial osteoblasts isolated from KO mice exhibit delayed rates of differentiation and defects in matrix production and mineralization [11]. Female TIEG1 KO mice also exhibit reduced osteocyte numbers with alterations in their ultra-structure [19]. Finally, we have demonstrated a reduction in the ability of TIEG1 KO osteoblasts to support osteoclast differentiation *in vitro* [11]. At the molecular level, loss of TIEG1 alters the expression of bone matrix genes as well as important osteoblast regulatory genes [11]. The expression of receptor activator of NFkB (RANKL), an inducer of osteoclastogenesis, is decreased in TIEG1 KO osteoblasts while the levels of osteoprotegerin (OPG), an inhibitor of osteoclastogenesis, are increased [11], [20]. These changes in expression of RANKL and OPG partially explain the loss of osteoblast support of osteoclastogenesis in these KO mice. As an extension of these studies, we have recently demonstrated that TIEG1 directly binds to GC-rich elements in the OPG promoter to suppress gene transcription [21]. However, the regulation of RANKL expression by TIEG1 appears to be indirect as no alterations in the activity of the RANKL promoter were observed in the presence or absence of TIEG1 expression [21].

Runx2 is known to be an important regulator of cell phenotype commitment and growth [22]–[26] and is considered a master gene in bone as it is essential for osteoblast lineage commitment, differentiation, bone matrix formation and mineralization [27]–[29]. Ablated or defective Runx2 expression in mice is lethal as there is an absence of osteoblasts and bone in such animal models [27], [30], [31]. Runx2 also plays a key role in mediating the BMP and TGFβ pathways which are important for osteoblast development and growth [32], [33]. Runx2 is required for normal expression of many genes known to be markers of a mature osteoblast including osteocalcin, type 1 collagen, ERα, Vit D receptor, alkaline phosphatase, bone sialo-protein, osteoprotegerin, osteopontin, collagenase, MCSF, BCL-2, RANKL, and osterix [34]–[42], [29]. As expected, tight regulation of Runx2 expression is necessary to ensure normal bone formation and maintenance as mice which over-express Runx2 specifically in osteoblasts also exhibit an osteopenic bone phenotype [43], [40]. Due to the importance of Runx2 in bone, identification of specific proteins that regulate Runx2 expression and function is essential to further our understanding of bone biology. Such genes and their associated pathways could serve as novel therapeutic targets for the treatment of numerous bone disorders including osteoporosis and fracture repair among others.

Based on the observed osteopenic phenotype of TIEG1 KO mice and the importance of Runx2 in bone, we sought to determine if alterations in Runx2 expression and/or function were observed in the absence of TIEG1. This study demonstrates that TIEG1 not only directly regulates Runx2 expression in osteoblasts, but also plays an active role in mediating Runx2 responses following TGFβ1 and BMP2 treatment. Additionally, the results indicate that TIEG1 associates with Runx2 protein and functions as a co-activator of Runx2 transcriptional activity. Further, decreased Runx2 expression in TIEG1 KO osteoblasts is at least in part responsible for their observed defects in differentiation and mineralization. Taken together, these data identify TIEG1 as a novel modulator of Runx2 expression and function and further implicate its importance in normal bone development and maintenance.

## Results

### Runx2 Expression in TIEG1 KO Osteoblasts

**Figure 1:**
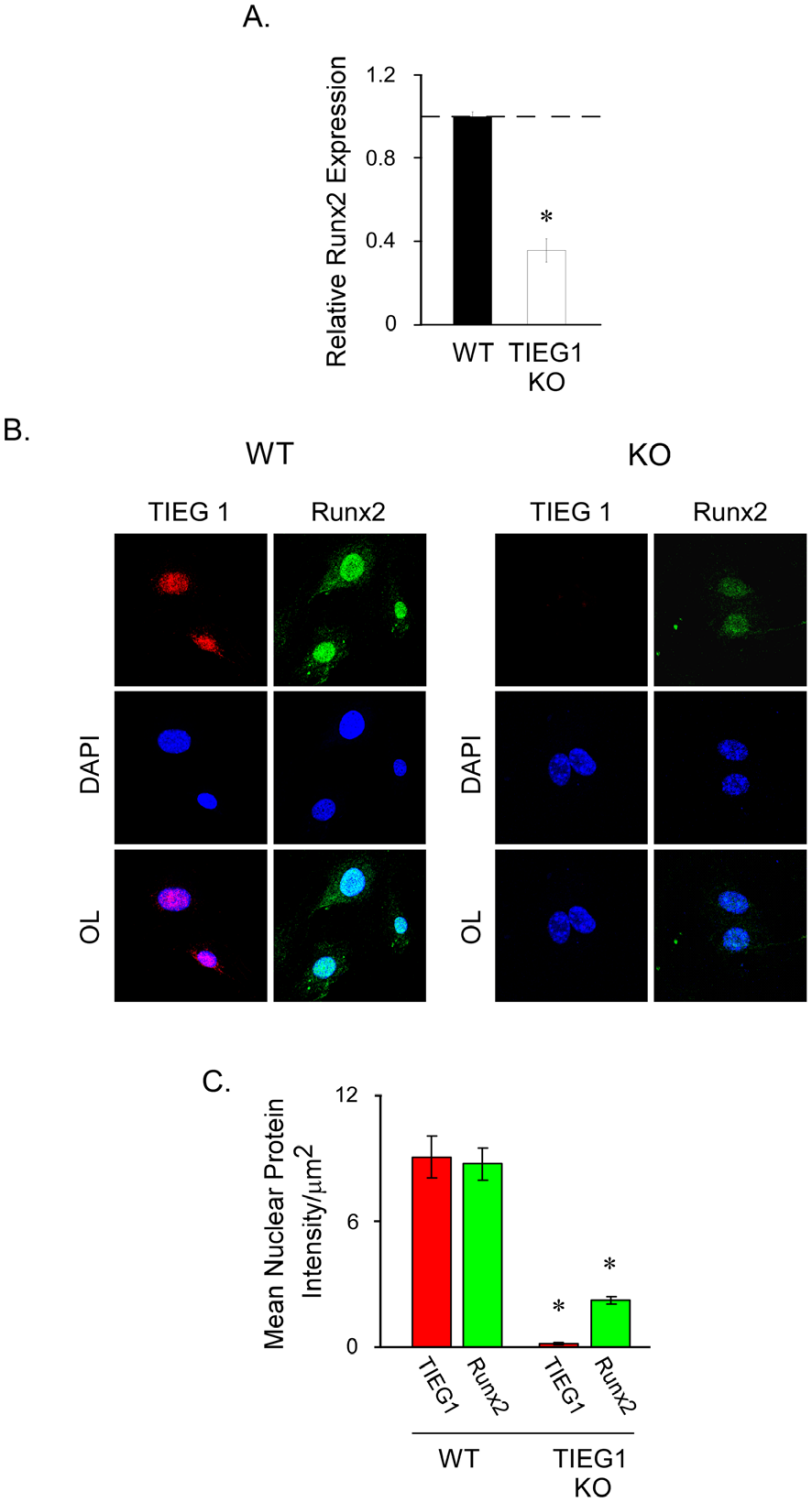
Runx2 expression levels are decreased in osteoblasts isolated from TIEG1 KO mice relative to WT controls. Calvarial osteoblasts were isolated from three wild-type (WT) and three TIEG1 knockout (KO) neonatal pups and cultured *in vitro*. (**A**) Total RNA was isolated from proliferating cells and real-time PCR analysis was performed to measure Runx2 mRNA levels. The results are depicted as relative expression levels compared to WT cells and represent average Runx2 expression across three distinct cell lines. (**B**) Representative confocal microscopy image depicting TIEG1 and Runx2 protein levels in WT and TIEG1 KO calvarial osteoblasts. (**C**) Quantitation of TIEG1 and Runx2 protein levels in WT and TIEG1 KO calvarial osteoblasts. *Asterisks* denote significance at the p<0.05 level (ANOVA) compared with WT cells.

This laboratory has previously demonstrated that calvarial osteoblasts isolated from TIEG1 KO mice exhibit reduced mineralization rates and decreased expression of a number of osteoblast marker genes relative to wildtype controls [11]. Due to the crucial role of Runx2 in mediating osteoblast differentiation and mineralization [27]–[29], it was of interest to quantify the expression levels of this gene in TIEG1 KO cells. Runx2 expression levels were analyzed in calvarial osteoblasts isolated from three individual wildtype and KO neonates via real-time RT-PCR. As shown in Figure 1A, Runx2 expression levels were significantly reduced in KO osteoblasts relative to wildtype littermates. Consistent with mRNA levels, nuclear Runx2 protein levels were also significantly repressed in KO cells as determined by quantitative confocal microscopy (Figure 1 B and C). These data suggest a role for TIEG1 in regulating the expression levels of this important osteogenic gene.

### Suppression of TIEG1 results in decreased Runx2 levels

**Figure 2:**
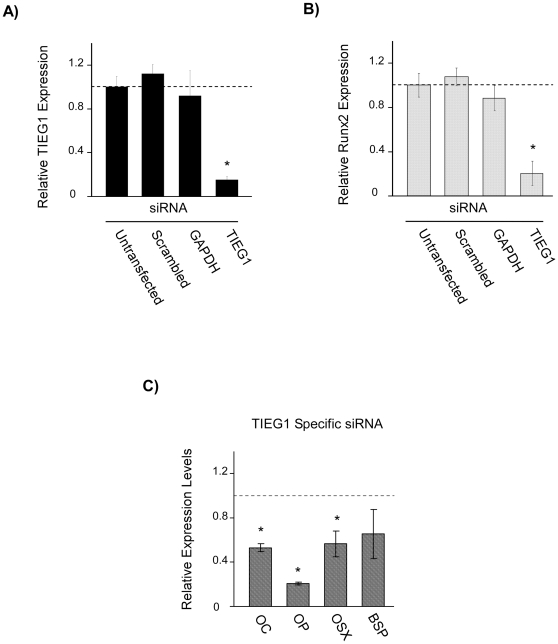
Suppression of TIEG1 in osteoblasts results in decreased expression of Runx2 and other osteoblast-related genes. Calvarial osteoblasts isolated from three wild-type neonatal pups were transfected with indicated siRNA constructs for 48 hours. Total RNA was isolated and TIEG1 (**A**) and Runx2 (**B**) expression levels were determined using real-time PCR. Data is reported as relative expression levels compared to untransfected cells. (**C**) The expression levels of osteocalcin (OC), osteopontin (OP), osterix (OSX) and bone sialoprotein (BSP) were also determined in WT calvarial osteoblasts transfected with the TIEG1 specific siRNA relative to cells transfected with the scrambled siRNA. *Asterisks* denote significance at the p<0.05 level (ANOVA) compared with indicated controls.

Since our TIEG1 mouse model is a global KO, we sought to determine if the effect of loss of TIEG1 expression on Runx2 levels was cell autonomous in osteoblasts. Wildtype calvarial osteoblasts were transfected with either non-sense (scrambled TIEG1 siRNA sequence), GAPDH or TIEG1 specific siRNA duplexes and the expression levels of TIEG1 and Runx2 were determined by real-time RT-PCR following normalization to untransfected control cells. TIEG1 expression was decreased by approximately 80% in cells transfected with the TIEG1 specific siRNA, but was unaltered in cells transfected with scrambled TIEG1 or GAPDH specific siRNAs (Figure 2A). Runx2 expression was also decreased by approximately 80% in cells transfected with TIEG1 siRNA, but not in cells transfected with non-sense or GAPDH siRNAs (Figure 2B). Since suppression of TIEG1 resulted in significant decreases in Runx2 expression, we next determined if this was associated with decreased expression of other osteoblast-related and Runx2 target genes. Indeed, the expression levels of osteocalcin, osteopontin, osterix and bone sialoprotein were also decreased in WT calvarial osteoblasts following transfection with a TIEG1 specific siRNA (Figure 2C). These studies provide further evidence that decreased expression of TIEG1 results in suppression of Runx2 and implicate TIEG1 as a novel regulator of Runx2 in osteoblasts. Furthermore, these studies provide evidence that decreased TIEG1 levels are also associated with decreased expression of other osteoblast-related genes, either as a result of direct regulation by TIEG1 or due to decreased expression of Runx2.

### Over-expression of TIEG1 results in increased Runx2 levels

**Figure 3:**
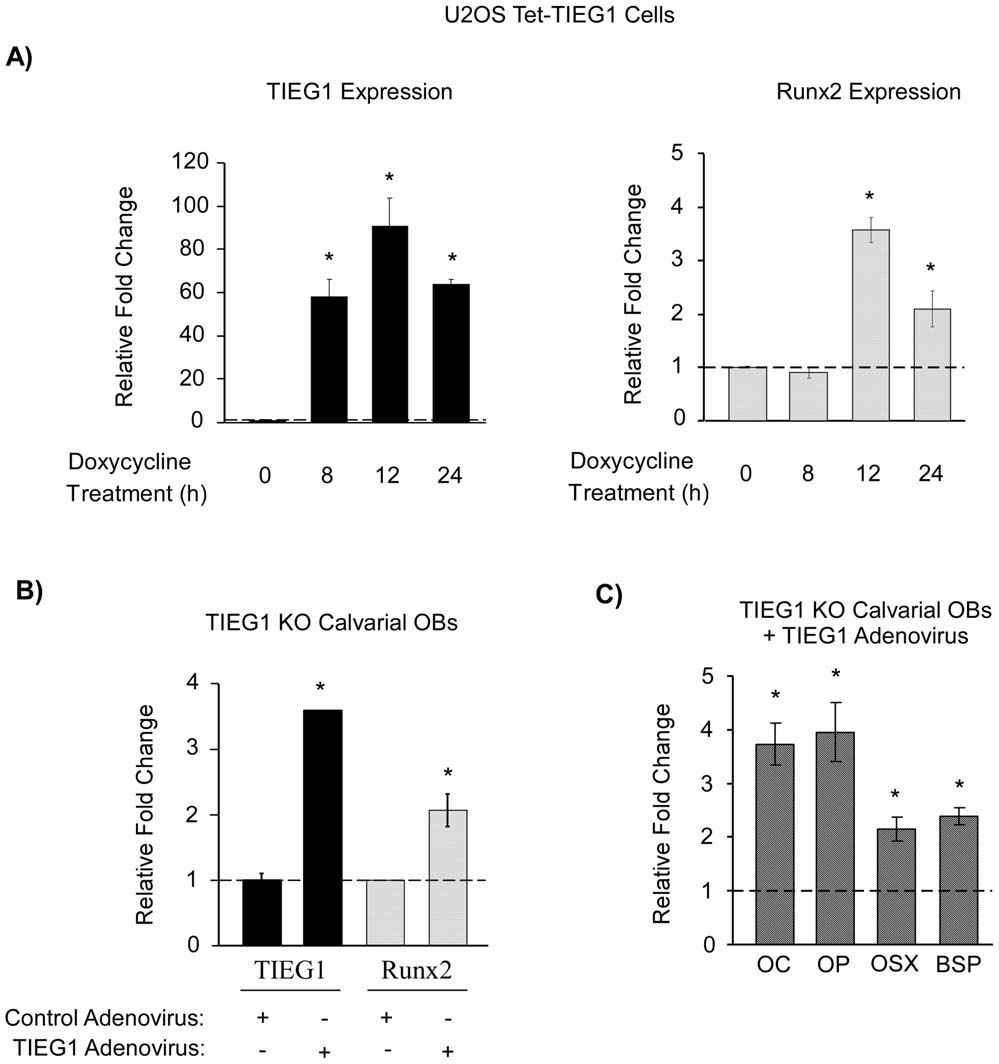
Over-expression of TIEG1 in osteoblasts results in increased expression of Runx2 and other osteoblast-related genes. (**A**) Inducible U2OS-TIEG1 cells (U2OS Tet-TIEG1) were treated with doxycycline for indicated times. Total RNA was harvested and TIEG1 and Runx2 expression levels were monitored by real-time PCR. (**B–C**) Calvarial osteoblasts isolated from three TIEG1 knockout (KO) neonatal pups were infected with either control or TIEG1 adenovirus for 24 hours. Total RNA was isolated and TIEG1 and Runx2 expression levels (**B**), as well as osteocalcin (OC), osteopontin (OP), osterix (OSX) and bone sialoprotein (BSP) (**C**), were determined using real-time PCR. The results are expressed as relative fold change compared to no doxycycline treatment (**A**) or to control adenoviral infected cells (**B–C**). *Asterisks* denote significance at the p<0.05 level (ANOVA) compared with controls.

As a complement to the siRNA studies, Runx2 expression levels were also determined in two different cell model systems in which TIEG1 was over-expressed. First, TIEG1 expression was activated in a U2OS-doxycycline inducible cell line (Tet-TIEG1) which was developed in our laboratory. TIEG1 expression was significantly elevated within 8 hours of doxycycline treatment, peaked at approximately 12 hours and was maintained at high levels at the 24 hour time point (Figure 3A). Subsequent to the induction of TIEG1 expression, Runx2 expression levels peaked at the 12 hour time point and remained elevated at 24 hours (Figure 3A).

We next determined if restoration of TIEG1 expression in calvarial osteoblasts isolated from TIEG1 KO mice would result in increased Runx2 expression levels. Infection of KO cells with a TIEG1 adenovirus resulted in an approximately 3.5 fold increase in TIEG1 expression levels (Figure 3B). As expected, Runx2 expression levels were also significantly elevated following induction of TIEG1 (Figure 3B). As a complement to the data shown in Figure 2C, we next determined if increased expression of TIEG1 and Runx2 were also associated with elevated levels of other osteoblast-related genes. As shown in Figure 3C, infection of KO calvarial osteoblasts with a TIEG1 adenovirus led to significant up-regulation of osteocalcin, osteopontin, osterix and bone sialoprotein expression. Taken together, these data reveal that increased expression of TIEG1 leads to induction of Runx2 and other osteoblast-related genes.

### TIEG1 mediates Runx2 responsiveness following TGFβ1 and BMP2 stimulation

**Figure 4:**
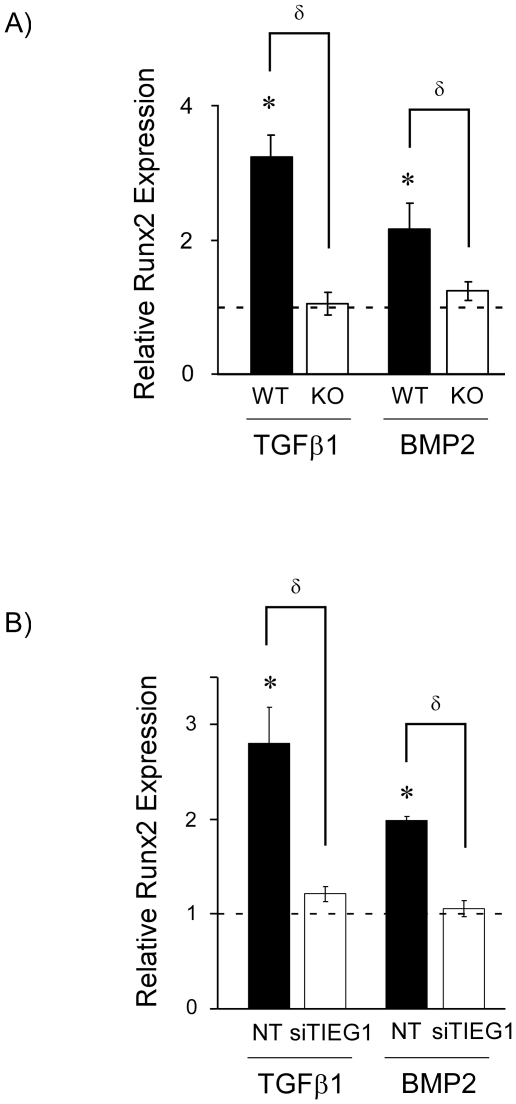
TIEG1 mediates Runx2 expression in osteoblasts following TGFβ1 and BMP2 stimulation. (**A**) Calvarial osteoblasts isolated from three wild-type (WT) and three TIEG1 knockout (KO) neonatal pups were treated with vehicle, TGFβ1 or BMP2 for 2 hours. Total RNA was isolated and Runx2 expression levels were monitored by real-time PCR. The results are expressed as relative fold change compared to vehicle treated cells and represent average Runx2 expression across three distinct cell lines. (**B**) WT calvarial osteoblasts were transfected with a scrambled (NT) or TIEG1 specific siRNA for 24 hours and subsequently treated with vehicle, TGFβ1 or BMP2 for 2 hours. Total RNA was isolated and Runx2 expression levels were determined by real-time PCR relative to vehicle controls. *Asterisks* denote significance at the p<0.05 level (ANOVA) compared to vehicle controls. δ denotes significance at the p<0.05 level (ANOVA) between WT and KO cells (**A**) or between WT cells transfected with either a scrambled (NT) or TIEG1 specific siRNA (**B**).

Since Runx2 expression is known to be induced within 2 hours by both TGFβ1 and BMP2 [32], and since TIEG1 expression is also rapidly induced by these two growth factors within 1 hour [44], [3], we next sought to determine if TIEG1 plays a role in mediating the induction of Runx2 by these two important bone anabolic cytokines. In order to monitor alterations in Runx2 expression in response to these cytokines as a result of TIEG1, three wild-type and three TIEG1 KO calvarial osteoblast cell lines were exposed to TGFβ1 or BMP2 for 2 hours and Runx2 expression levels were monitored by real-time PCR. As expected, Runx2 expression was rapidly induced by both TGFβ1 and BMP2 in wildtype cells (Figure 4A). However, this induction was nearly abolished in TIEG1 KO cells (Figure 4A) suggesting that TIEG1 plays a role in mediating the response of Runx2 expression in osteoblasts following TGFβ1 and BMP2 stimulation.

As an extension of these studies, we next determined if restoration of TIEG1 expression in KO calvarial osteoblasts restored the ability of TGFβ1 and BMP2 to induce Runx2 expression. As expected, over-expression of TIEG1 in KO cells led to increased expression of Runx2, however, no further increases in Runx2 expression levels were observed in response to TGFβ1 or BMP2 treatment (data not shown). This observation suggested that the induction of TIEG1 expression by TGFβ1 and BMP2 was an essential component in the cascade of events leading to up-regulation of Runx2 by these two cytokines. In order to address this possibility, we next suppressed TIEG1 expression in WT calvarial osteoblasts using a TIEG1 specific siRNA and subsequently monitored Runx2 expression levels following 2 hours of TGFβ1 or BMP2 treatment. Interestingly, suppression of TIEG1 prevented the induction of Runx2 expression following TGFβ1 and BMP2 treatment (Figure 4B). However, these two cytokines induced Runx2 expression in WT cells transfected with a scrambled (NT) siRNA (Figure 4B). Taken together, these data suggest that increased expression of TIEG1 following TGFβ1 and BMP2 stimulation is necessary for the rapid induction of Runx2 expression by these two cytokines in osteoblast cells.

### TIEG1 activates the Runx2 promoter

**Figure 5:**
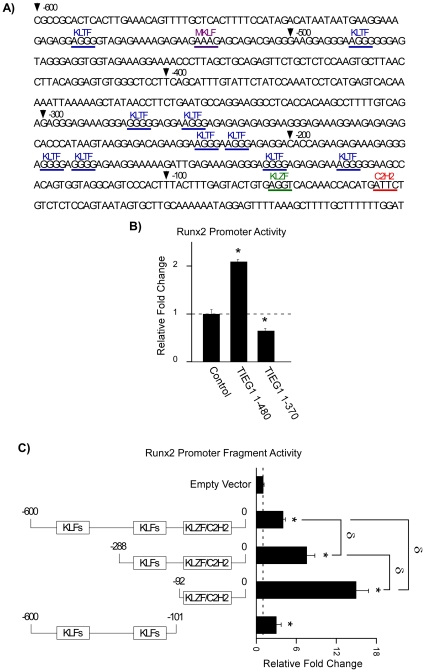
Regulation of Runx2 promoter activity by TIEG1 in osteoblasts. (**A**) Putative krüppel-like transcription factor binding sites located within the −600 bp fragment of the Runx2 P1 promoter as identified using the Genomatix software suite. Krüppel-like transcription factor (KLTF), mouse krüppel like factor (MKLF), krüppel like zinc finger (KLZF) and C2H2 zinc finger containing transcription factor (C2H2) binding sites are indicated. (**B**) U2OS cells were transiently transfected with indicated control, full-length TIEG1 (1–480) or truncated TIEG1 (1–370) expression vectors and the full-length Runx2 promoter (−600) fused to a luciferase reporter. (**C**) U2OS cells were transiently transfected with indicated control or Runx2 promoter constructs fused to a luciferase reporter along with the full-length (1–480) TIEG1 expression construct. Twenty four hours post-transfection, luciferase activity was monitored and values are reported as relative fold change compared to controls following normalization to total protein levels. *Asterisks* denote significance at the p<0.05 level (ANOVA) compared with control values. δ denotes significance at the p<0.05 level (ANOVA) between indicated promoter constructs.

Since the above data support a role for TIEG1 in regulating Runx2 expression, we next determined if TIEG1 could activate the Runx2 promoter. As a first step, an approximately 600 base pair fragment of the Runx2 P1 promoter [45] was analyzed for potential TIEG1 binding sites using the Genomatix software suite (Munich, Germany). This transcription factor binding site search identified multiple putative krüppel-like transcription factor binding sites which are indicated in Figure 5A. This promoter construct was subsequently transfected into U2OS cells with a control expression vector, a full-length TIEG1 expression vector or a truncated TIEG1 expression vector in which the DNA binding domain was deleted (TIEG1 1–370) to determine if TIEG1 could regulate its activity. As shown in Figure 5B, intact TIEG1 significantly induced Runx2 promoter activity. DNA binding by TIEG1 was shown to be essential for this activation since deletion of the zinc-finger containing domain rendered this protein inactive on the Runx2 promoter (Figure 5B). To further define the region of the Runx2 promoter through which TIEG1 functions, 5′-deletion constructs of the promoter [45] were transfected into U2OS cells with either a control or full-length TIEG1 expression vector. All of the promoter constructs analyzed were significantly induced by TIEG1, and the levels of induction progressively increased as the 5′-region of the Runx2 promoter was deleted (Figure 5C). Since the −92 bp fragment of the Runx2 promoter appeared to be the most active, this region was then deleted in the context of the full-length promoter. Deletion of this 3′-region did not completely abolish the TIEG1 induction of promoter activity, and in fact, the activity of this construct was nearly identical to that of the intact −600 bp promoter (Figure 5C). These data indicate that TIEG1 likely utilizes multiple binding sites located throughout the Runx2 promoter and furthermore suggest that repressive domains, which reduce the ability of TIEG1 to activate this promoter, are contained within the distal regions of the Runx2 P1 promoter.

### TIEG1 stabilization further enhances Runx2 promoter activity

**Figure 6:**
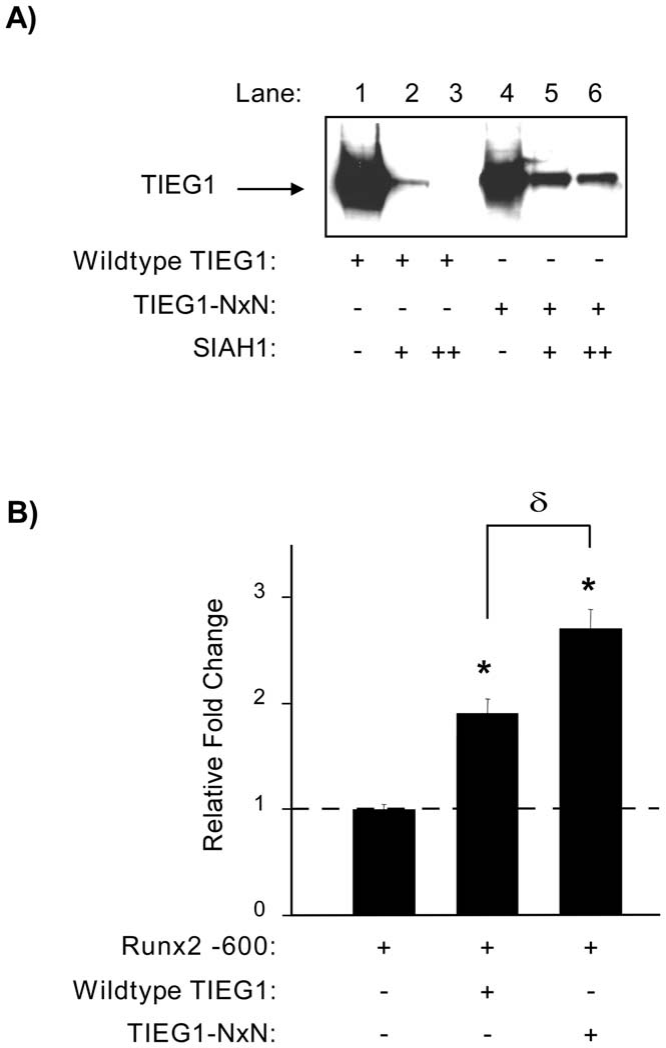
Stabilization of TIEG1 protein levels result in enhancement of Runx2 promoter activity. (**A**) Western blot analysis depicting wild-type TIEG1 and TIEG1-NxN protein levels following co-transfection with increasing amounts of a SIAH1 expression construct in U2OS cells using a Flag-specific primary antibody. (**B**) Indicated TIEG1 expression vectors and the full-length Runx2 promoter (−600) reporter construct were transiently transfected into U2OS cells. Twenty four hours post-transfection, luciferase activity was monitored and values are reported as relative fold change compared to controls. *Asterisks* denote significance at the p<0.05 level (ANOVA) compared with control values. δ denotes significance at the p<0.05 level (ANOVA) between wild-type and NxN TIEG1 expression constructs.

Our laboratory has previously reported that TIEG1 expression and function is tightly regulated by the ubiquitin/proteasome system as TIEG1 protein is a direct target for the E3 ubiquitin ligase, seven in absentia homolog 1 (SIAH1) [46]. The SIAH1 binding site for TIEG1 has been mapped [47] and mutation of valine 205 and proline 207 to asparagines (TIEG1-NxN) results in stabilization of this protein. Based on these previous observations, we examined whether this pathway may be involved in modulating Runx2 expression as a result of TIEG1 stability. As expected, increased expression of SIAH1 in U2OS cells completely abolished TIEG1 protein levels (Figure 6A). However, co-transfection of the TIEG1-NxN expression construct resulted in significant stabilization of this protein in the presence of SIAH1 (Figure 6A). Stabilization of TIEG1 protein levels (TIEG1-NxN) resulted in further activation of the −600 Runx2 promoter construct relative to WT TIEG1 (Figure 6B). While these data do not directly implicate a role for SIAH1 in modulating Runx2 expression via TIEG1 stability, they do suggest that the ubiquitin/proteasome pathway may be involved in fine tuning the expression levels of Runx2 in osteoblasts through mediating TIEG1 protein levels.

### TIEG1 directly binds to the Runx2 promoter

**Figure 7:**
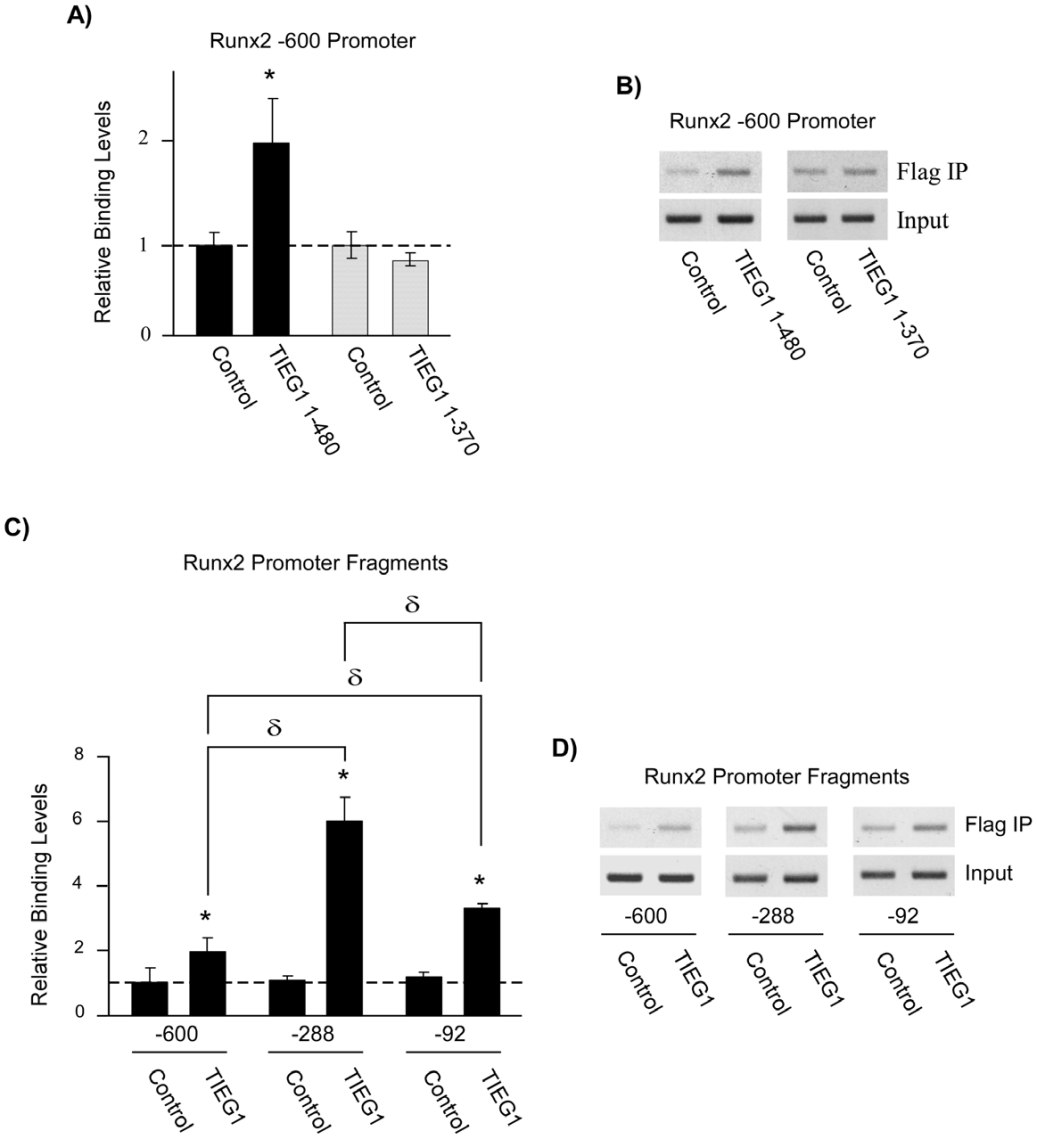
TIEG1 associates with the Runx2 promoter in a DNA binding dependent manner. (**A–D**) Transient chromatin immunoprecipitation (ChIP) assays were performed in U2OS cells transfected with indicated Flag-tagged expression vectors and promoter constructs for 24 hours. Chromatin was prepared, immunoprecipitated with a Flag specific antibody and amplified by both real-time PCR (**A and C**) and semi-quantitative PCR (**B and D**). Real-time PCR analysis was utilized for quantitation purposes and the data are expressed as the abundance of the Runx2 promoter relative to cells transfected with a control expression construct. All data were normalized using input samples. *Asterisks* denote significance at the p<0.05 level (ANOVA) compared with controls. δ denotes significance at the p<0.05 level (ANOVA) between indicated promoter constructs. The products obtained by semi-quantitative PCR were separated using agarose gel electrophoresis.

Based on the previous data indicating a role for TIEG1 in regulating Runx2 expression, we next sought to determine if TIEG1 protein directly binds to the Runx2 promoter. As a first step, the Runx2 −600 promoter construct was co-transfected into U2OS cells with either a control, full-length TIEG1 or truncated TIEG1 (TIEG1 1–370) expression vector. Chromatin immunoprecipitation (ChIP) assays revealed increased binding of full-length TIEG1, but not truncated TIEG1 (TIEG1 1–370), above control levels (Figure 7A and B) demonstrating that TIEG1 does associate with the Runx2 promoter in a DNA binding dependent manner. TIEG1 binding was further enhanced on the Runx2 −288 and −92 promoter constructs relative to the full-length promoter construct with maximal levels of binding observed on the Runx2 −288 promoter fragment (Figure 7C and D). These data correlate well with the potential TIEG1 binding sites indentified in the Runx2 promoter as shown in Figure 5A since 10 of the 13 putative TIEG1 binding sites are contained within the −288 fragment and only 2 remain in the −92 fragment. However, the −92 bp fragment was the most highly activated construct by TIEG1 at the transcriptional level (Figure 5C) suggesting that repression domains are located between −288 and −92 bps which inhibit optimal activation of the Runx2 promoter by TIEG1. The basis for decreased binding of TIEG1 to the −600 bp construct is also likely explained by the presence of inhibitory factors/repressive domains located in the distal regions of the Runx2 promoter that block TIEG1's ability to bind to and/or optimally activate this promoter fragment. Nevertheless, these data reveal that TIEG1 specifically interacts with the Runx2 promoter in a DNA binding dependent manner and further confirm a role for TIEG1 in regulating Runx2 expression.

### TIEG1 interacts with Runx2 protein and regulates its activity

**Figure 8:**
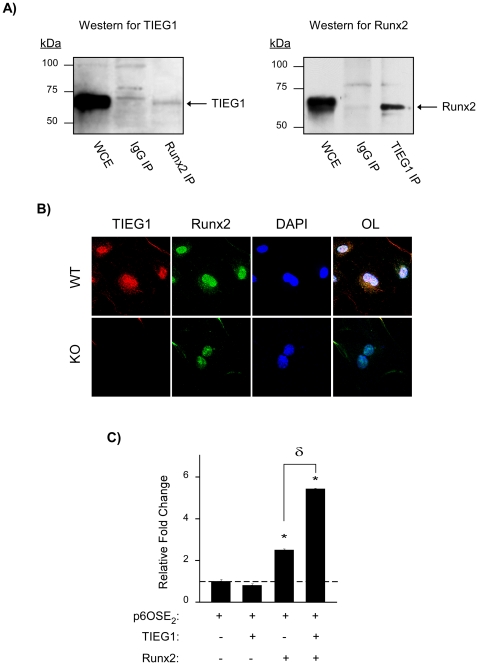
TIEG1 interacts with and co-activates Runx2 transcriptional activity in osteoblasts. (**A**) U2OS cells were co-transfected with TIEG1 and Runx2 expression constructs. Cells were lysed and equal amounts of protein were immunoprecipitated with either a Runx2 or TIEG1 specific antibody as well as an IgG control antibody. Immunoprecipitated protein complexes were separated by SDS-PAGE and western blotting was performed using either a TIEG1 or Runx2 specific antibody. Whole cell extracts (WCE) were loaded as positive controls. Arrows indicate TIEG1 and Runx2 protein following immunoprecipitation. (**B**) Representative confocal microscopy image depicting co-localization of TIEG1 and Runx2 protein in wild-type (WT), but not TIEG1 knockout (KO), calvarial osteoblasts. (**C**) U2OS cells were transiently transfected with a p6OSE_2_ luciferase reporter construct and TIEG1 or Runx2 expression vectors as indicated. Twenty four hours post-transfection, luciferase activity was monitored and values are reported as relative fold change compared to empty vector controls. *Asterisks* denote significance at the p<0.05 level (ANOVA) compared with control values. δ denotes significance at the p<0.05 level (ANOVA) between cells transfected with Runx2 alone and cells transfected with both Runx2 and TIEG1.

Since TIEG1 and Runx2 are both transcription factors which are known to regulate multiple bone related genes, and since we have demonstrated that TIEG1 directly regulates Runx2 expression, we next examined whether TIEG1 could interact with the Runx2 protein and mediate its activity. U2OS cells were first co-transfected with TIEG1 and Runx2 expression constructs for 24 hours. Cell lysates were immunoprecipitated with normal rabbit IgG, a Runx2 specific antibody or a TIEG1 specific antibody followed by western blotting with reciprocal antibodies. As shown in Figure 8A, TIEG1 protein was immunoprecipitated with the Runx2 specific antibody, and Runx2 protein was immunoprecipitated with the TIEG1 specific antibody. Neither protein was immunoprecipitated with the non-specific IgG control antibody (Figure 8A). These data demonstrate that these two proteins specifically associate with each other in a protein complex. Furthermore, TIEG1 and Runx2 protein were shown to co-localize in the nucleus of WT calvarial osteoblasts via confocal microscopy providing further support for the interaction of these two transcription factors in osteoblasts (Figure 8B). In order to determine if this interaction affected Runx2 transcriptional activity, U2OS cells were next transfected with a known Runx2-responsive luciferase reporter construct, p6OSE2-Luc [48], and either a TIEG1 or Runx2 expression construct, alone or in combination. As expected, Runx2 induced p6OSE_2_ activity; however TIEG1 alone had no effect on this enhancer element (Figure 8C). Importantly, co-expression of TIEG1 and Runx2 led to co-activation of this reporter construct (Figure 8C). In combination, these data demonstrate that TIEG1 and Runx2 proteins are able to associate with one another in a complex leading to increased Runx2 transcriptional activity in osteoblasts.

### Increased expression of Runx2 in TIEG1 KO osteoblasts partially rescues their mineralization defects

**Figure 9:**
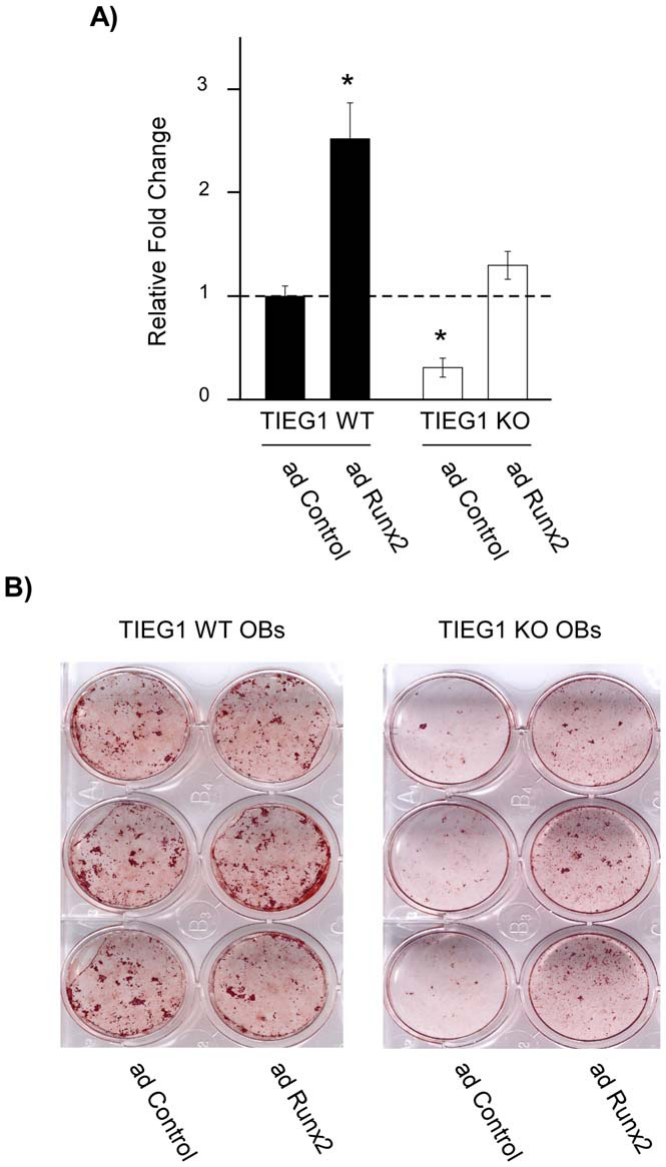
Restoration of Runx2 expression in TIEG1 KO osteoblasts partially rescues their differentiation and mineralization defects. (**A**) Calvarial osteoblasts isolated from three wild-type (WT) and three TIEG1 knockout (KO) neonatal pups were infected with either a control or Runx2 adenovirus for 24 hours. Total RNA was isolated and Runx2 expression levels were determined using real-time PCR. The results are expressed as relative fold change compared to control adenoviral infected cells and represent average Runx2 expression across three distinct cell lines. *Asterisks* denote significance at the p<0.05 level (ANOVA) compared with WT control. (**B**) Three WT and three TIEG1 KO calvarial osteoblast cell lines were plated in 6-well plates and allowed to proliferate to confluence. Once confluent, cells were cultured in osteoblastic differentiation medium in the presence of a control or Runx2 adenovirus as indicated for 21 days. Following differentiation, cells were stained with alizarin red and a representative wild-type and TIEG1 KO cell line is shown.

This laboratory has previously shown that calvarial osteoblasts isolated from TIEG1 KO mice exhibit significant delays in differentiation and a reduced ability to mineralize matrix [11]. This defect is associated with decreased expression of multiple bone related genes [11]. Based on the present study in which we have demonstrated that TIEG1 directly regulates both the expression and activity of Runx2, it was of interest to determine if increased Runx2 levels could rescue the mineralization defects observed in TIEG1 KO calvarial osteoblasts. Three WT and three TIEG1 KO cell lines were infected with either a control or Runx2 adenovirus and differentiation assays were performed over the course of 21 days. As shown in Figure 9A, Runx2 expression levels were significantly increased in WT and KO cells following infection with a Runx2 adenovirus relative to control adenovirus. Runx2 expression was increased in KO cells to levels comparable to that of endogenous Runx2 expression in WT cells (Figure 9A). Increased expression of Runx2 in WT cells had no effect on their mineralization capacity (Figure 9B) suggesting that the TIEG1/Runx2 axis was functioning maximally in these cells. However, restoration of Runx2 expression in TIEG1 KO cells partially rescued their mineralization defect (Figure 9B). These data suggest that decreased expression of Runx2 in TIEG1 KO cells is at least in part responsible for their reduced mineralization capacity and imply an important role for Runx2 in the observed osteopenic phenotype previously described in these mice [17], [18]. However, other important osteoblast related genes and/or pathways are likely implicated in this defect since restoration of Runx2 expression in TIEG1 KO cells did not completely restore their mineralization capacity to that of WT osteoblasts.

## Discussion

**Figure 10:**
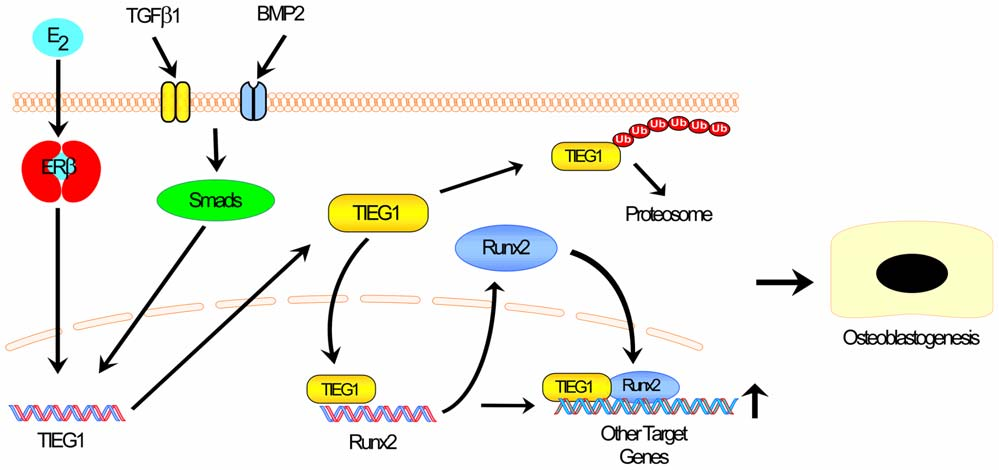
Model depicting the mechanisms by which TIEG1 mediates Runx2 expression and activity in osteoblasts.

This study demonstrates that suppression of TIEG1 results in decreased expression of Runx2 while over-expression of TIEG1 up-regulates Runx2 levels. Runx2 promoter luciferase reporter constructs and ChIP analyses have revealed that TIEG1's regulation of Runx2 expression occurs in a DNA binding dependent manner through regulatory elements located in the proximal region of the P1 promoter. We have also provided evidence that TIEG1 plays a role in mediating the responsiveness and magnitude of Runx2 expression following TGFβ1 and BMP2 treatment and have implicated the ubiquitin/proteasome pathway as another mechanism to precisely control Runx2 levels by targeting TIEG1 for degradation. Furthermore, we have shown that TIEG1 can interact with the Runx2 protein and serve as a co-activator for Runx2 transcriptional activity. Finally, osteoblast differentiation studies have demonstrated that restoration of Runx2 expression in TIEG1 KO cells partially rescues their mineralization defect implicating a role for this signaling pathway in mediating the osteopenic phenotype of TIEG1 KO mice [17], [18]. Therefore, a model has evolved whereby TIEG1 serves as an important mediator of both Runx2 expression levels and Runx2 transcriptional activity in osteoblasts (Figure 10).

The importance of Runx2 with regard to skeletal development and osteoblast differentiation has been well described. Runx2 plays a critical role as a lineage determining transcription factor which is expressed in mesenchymal precursor cells and functions to direct their differentiation into osteoblasts [49]. Runx2 is also considered a master regulator of osteoblast differentiation as it induces the expression of osterix, another transcription factor whose expression is essential for terminal osteoblast differentiation and mineralization [50]. In addition to osterix, Runx2 regulates many other osteoblast related genes [27], [30]. Deletion of Runx2 in mice causes arrest of osteoblast differentiation and results in neonates with a completely cartilaginous skeleton which die shortly after birth [34], [27], [30]. Furthermore, germline mutations in the Runx2 gene are strongly associated with patients diagnosed with cleidocranial dysplasia, an autosomal dominant skeletal disorder [51].

The precise regulation of Runx2 expression and activity is essential for normal bone formation as mice over-expressing Runx2 under the control of the collagen 1a1 2.3-kb promoter exhibit an osteopenic phenotype [43], [40]. Indeed, Runx2 has been shown to be regulated at the level of transcriptional control, protein activity and protein turnover. However, the specific factors and their associated signaling pathways involved in these processes continue to be elucidated. At the level of transcriptional control, a small number of transcription factors have been indentified which directly regulate Runx2 expression levels. Positive regulators of Runx2 expression in osteoblasts include the homeobox genes, Msx2 [52] and Bapx1 [53], as well as RBP1 [54], SP1 and ELK1 [55]. In contrast, Hoax2 [56] and Sox8 [57] have been shown to inhibit Runx2 expression. In the present study, TIEG1 is shown to act as a positive regulator of Runx2 expression in osteoblasts implying an additional role for this transcription factor in regulating osteoblastogenesis and bone development through the actions of Runx2.

At the level of Runx2 function, many proteins have been identified that interact with this important transcription factor to modulate its activity. Proteins such as Stat1 [58], Sox9 [59], Aj18 [60], MEF [61], Nrf2 [62], YAP [63], HDAC4 [64], and p53 [65] have all been shown to interact with the Runx2 protein to inhibit its transcriptional activity. Positive regulators of Runx2 transcriptional activity include CBFβ1 [66]–[68], Smads 1 and 5 [32], [69], Satb2 [56], Rb [70], TAZ [71], HOXA10 [72], BAPX-1 [53], RBP1 [54], C/EBPβ, C/EBPδ and Menin [73], among others. This manuscript demonstrates that TIEG1 also interacts with the Runx2 protein in osteoblast cells and serves as a co-activator of Runx2 transcriptional activity.

In addition to individual genes and proteins affecting Runx2 expression and activity, a number of cytokines have been shown to influence Runx2 mRNA levels. Specifically, TGFβ1 and BMP2 treatment of osteoblast cells results in the rapid up-regulation or Runx2 expression [32]. Because TIEG1 was originally identified as a primary response gene following TGFβ1 treatment [1], and was later shown to be induced by BMP2 [3], we speculated that it may play a role in mediating Runx2 expression by these two factors. Indeed, Runx2 responsiveness to TGFβ1 and BMP2 is suppressed in osteoblasts isolated from TIEG1 KO mice. Furthermore, TGFβ1 and BMP2 do not lead to up-regulation of Runx2 expression in WT cells transfected with a TIEG1 specific siRNA. These data suggest that TIEG1 functions upstream of Runx2 in the cascade of events that are initiated by TGFβ1 and BMP2 and implicate a role for TIEG1 in the optimal and immediate responsiveness of Runx2 to these cytokines in bone cells.

A final level controlling Runx2 function in osteoblasts is through the targeted degradation of Runx2 protein. Shn3 is a zinc finger containing adapter protein that links Runx2 with the E3 ubiquitin ligase, WWP1 [74]. This interaction ultimately results in poly-ubiquitination of Runx2 followed by proteasomal degradation. Notably, TIEG1 is also highly targeted by another E3 ubiquitin ligase family member, SIAH1 [46]. Here, we have demonstrated that inhibiting the interaction between SIAH1 and TIEG1 results in further activation of the Runx2 promoter. Since we have also shown that TIEG1 and Runx2 proteins interact, one could speculate that complexes consisting of these two proteins could be rapidly targeted, together or separately, for proteasomal degradation. Regardless, these data further implicate the ubiquitin/proteasome system in the regulation of Runx2 levels both through directly targeting Runx2 protein for degradation, and by targeting TIEG1, an inducer of Runx2 expression, for degradation. This cellular process further serves to tightly regulate the molecular actions of these two important bone related transcription factors.

The studies presented in this manuscript identify TIEG1 as a novel and important regulator of Runx2 expression and activity. TIEG1 serves as both an inducer of Runx2 expression, as well as a co-activator of Runx2 transcriptional activity. TIEG1 is also shown to play an important role in mediating Runx2 responses to cytokines in osteoblast cells. The ubiquitin/proteasome system has been implicated as another mechanism to control the levels of Runx2 by regulating TIEG1 protein stability. Finally, a partial role for Runx2 has been identified in mediating the defects in osteoblast differentiation and mineralization observed in TIEG1 KO mice. These observations add another dimension to the tightly controlled and critical regulation of Runx2 expression and function in osteoblasts and identify TIEG1 as a potential target for approaches to treat bone related diseases resulting from defects in any number of the cellular pathways presented here.

## Materials and Methods

### Cell Culture and Calvarial Osteoblast Isolation

Calvarial osteoblasts were isolated from 3 day old WT or TIEG1 KO neonatal pups obtained from heterozygous breeding pairs as described previously [11]. Following isolation, cells were maintained in α-MEM (Invitrogen, Carlsbad, CA) containing 10% (v/v) fetal bovine serum (FBS) (ISC Bioexpress, Kaysville, UT) and 1% (v/v) antibiotic/antimycotic (Invitrogen, Carlsbad, CA) in a humidified 37°C incubator with 5% CO_2_. All experiments involving calvarial osteoblasts were conducted within the first two passages from the time of isolation. This study was carried out in strict accordance with the recommendations in the Guide for the Care and Use of Laboratory Animals of the National Institutes of Health. The protocol was approved by the Mayo Clinic Institutional Animal Care and Use Committee (Permit Number: A9709).

Parental U2OS cells were purchased from ATCC. The U2OS-TIEG1 doxycycline inducible cell line (U2OS Tet-TIEG1) was developed using parental U2OS cells and the T-Rex system (Invitrogen) according to the manufacturer's protocol. Cells were cultured in phenol red-free Dulbecco's modified Eagle's medium/F12 medium (DMEM/F12) containing 10% (v/v) FBS, 1% (v/v) antibiotic/antimycotic, 5 mg/liter blasticidin S (Roche Applied Science, Indianapolis, IN), and 500 mg/liter zeocin (Invitrogen). Parental U2OS cells were cultured in DMEM/F12 containing 10% (v/v) FBS and 1% (v/v) antibiotic/antimycotic solution.

### RNA Isolation and Real-Time PCR Analysis

Wildtype and TIEG1 KO calvarial osteoblasts were plated in 12 well plates at a density of approximately 50%. To account for individual variability of these primary cell lines, all studies were performed in triplicate using osteoblasts isolated from three different WT and TIEG1 KO pups. Cells were allowed to proliferate until they were approximately 80% confluent at which time they were washed twice with 1x PBS. Total RNA was isolated using Trizol reagent (Invitrogen) as specified by the manufacturer. RNA yield was determined using a NanoDrop 1000 spectrophotometer (Thermo Fisher Scientific, Wilmington, DE).

Five hundred ng of total RNA was reverse transcribed using the iScript™ cDNA Synthesis Kit (Bio-Rad, Hercules, CA). Real-time PCR was performed in triplicate using a Bio-Rad iCycler and a PerfeCTa™ SYBR Green Fast Mix™ for iQ real-time PCR kit (Quanta Biosciences, Gaithersburg, MD) as specified by the manufacturer. Cycling conditions were as follows: 95°C for 2 minutes followed by 40 cycles of 95°C for 1 second and 60°C for 30 seconds. Melt curves were generated to ensure amplification of a single PCR product. Quantitation of the PCR results were calculated based on the threshold cycle (C_t_) and were normalized using β-tubulin for all mouse transcripts or TATA Binding Protein (TBP) for all human transcripts. All PCR primers were designed using Primer3 software (http://frodo.wi.mit.edu/primer3/) and were purchased from Integrated DNA Technologies (Coralville, IA). Primer sequences are listed in Table 1.

**Table 1:** Primer sets used in qRT-PCR and ChIP assays.

	Primer, 5′ - 3′	
Gene	Forward	Reverse
mRunx2	GCCGGGAATGATGAGAACTA	GGTGAAACTCTTGCCTCGTC
mOsteocalcin	GCCATCACCCTGTCTCCTAA	GCTGTGGAGAAGACACACGA
mOsteopontin	CCCGGTGAAAGTGACTGATTCT	GATCTGGGTGCAGGCTGTAAA
mOsterix	GGAGGTTTCACTCCATTCCA	TAGAAGGAGCAAGGGGACAGA
mBone Sialoprotein	TTCCCAGGTGTGTCATTGAAGA	GGTATGTTTGCGCAGTTAGCAA
mKLF10	GTGACCGTCGGTTTATGAGG	ACTTCCATTTGCCAGTTTGG
hRunx2	CCAGATGGGACTGTGGTTACTG	TTCCGGAGCTCAGCAGAATAA
hKLF10	GCCAACCATGCTCAACTTCG	TGCAGTTTTGTTCCAGGAATACAT
mβ-Tubulin	CTGCTCATCAGCAAGATCAGAG	GCATTATAGGGCTCCACCACAG
hTBP	AGTTGTACAGAAGTTGGGTTTTC	AACAATTCTGGGTTTGATCATTC
Runx2 ChIP	AATAGTGCTTGCAAAAAATAGGAGTTT	TGGCGTCTTCCATGGTGGCTTTAC

### Confocal Microscopy

Localization and quantitation of TIEG1 and Runx2 protein in WT and TIEG1 KO calvarial osteoblasts were determined using immunoflorescence histochemistry with a Zeiss LSM 510 confocal microscope (Carl Zeiss, Jena, Germany). Briefly, cells were fixed in 1% paraformaldehyde for 30 min and washed twice with 1X PBS. Cells were permeabilized with 0.2% Triton-X in PBS for 30 min and blocked for an additional 30 min in heat-inactivated 5% FBS. Subsequently, cells were incubated with a polyclonal TIEG1 antibody produced by our laboratory (PAb # 992) and a monoclonal Runx2 antibody (clone # 8G5, Medical and Biological Laboratories, Naka-ku Nagoya, Japan) for 60 min. Cells were washed twice with PBS and stained with Texas Red- and FITC-conjugated secondary IgG Antibodies (Santa Cruz Biotechnology, Santa Cruz, CA) for an additional 60 minutes. DAPI was used as a counter stain. Relative TIEG1 and Runx2 protein levels were quantified using Zeiss LSM 510 software.

### TIEG1 Specific siRNAs

Wildtype calvarial osteoblasts were cultured as above and transfected with either a TIEG1 specific siRNA (AAUGGAACUAAUUUCUGAA)d(TT)), a scrambled TIEG1 siRNA (non-sense) or a GAPDH specific siRNA. TIEG1 and non-sense siRNAs were custom designed and purchased from Darmacon (Lafayette, CO) while GAPDH specific siRNA was purchased from Ambion (Austin, TX). Transient transfections were performed using HiPerfect transfection reagent (Qiagen, Valencia, CA) according to the manufacturer's protocol. Forty eight hours post-transfection, total RNA was isolated and reverse transcribed. TIEG1, and Runx2 expression levels were determined via real-time PCR in each of the transfected cultures relative to non-transfected control cells. Osteocalcin, osteopontin, osterix and bone sialoprotein expression levels were quantitated in WT calvarial osteoblasts transfected with the TIEG1 specific siRNA relative to cells transfected with the scrambled siRNA.

### TIEG1 Over-Expression in U2OS Osteosarcoma Cells

U2OS Tet-TIEG1 cells were plated at a density of approximately 50% in triplicate in 12 well tissue culture plates. The following day, cells were treated with 100 ng/mL of doxycycline for 0, 8, 12 or 24 hours. Total RNA was isolated using Trizol and reverse transcribed as specified above. TIEG1 and Runx2 expression levels were monitored via real-time PCR.

### TIEG1 and Runx2 Adenovirus

The mouse TIEG1 coding sequence was cloned into the adenoviral-Type 5 (dE1/E3) vector and adenoviral production, amplification, purification and titer determination was conducted by Vector Biolabs (Philadelphia, PA). A control adenovirus containing an empty expression vector was also supplied by Vector Biolabs. The Runx2 adenovirus was provided by Dr. Gary Stein's laboratory. For TIEG1 overexpression, 50% confluent TIEG1 KO calvarial osteoblasts were infected at a multiplicity of infection (MOI) of 10 for 24 hours. Subsequently, the expression levels of TIEG1, Runx2, osteocalcin, osteopontin, osterix and bone sialoprotein were determined via real-time PCR. For Runx2 overexpression in differentiating calvarial osteoblasts, confluent WT and TIEG1 KO cells were infected at a MOI of 10 in osteoblast differentiation medium (MEMα +10% FBS +1% antibiotic-antimycotic +50 mg/L ascorbic acid +10 mM β-glycerophosphate). Differentiation medium was changed every 3 days and fresh adenovirus was added with each feeding over the course of 21 days at which time cells were fixed and stained with alizarin red.

### TGFβ1 and BMP2 Treatments

Wildtype and TIEG1 KO calvarial osteoblasts were plated in triplicate in 12 well tissue culture plates at a density of approximately 50%. The following day, cells were treated with vehicle (0.25% BSA in 1X PBS), TGFβ1 (2 ng/mL) or BMP2 (200 ng/mL) for 2 hours. TGFβ1 was purchased from Austral Biologicals (San Ramon, CA) while BMP2 was purchased from R&D Systems (Minneapolis, MN). Total RNA was harvested, cDNA was prepared and real-time PCR was conducted to determine Runx2 expression levels as described above. Additionally, WT and KO calvarial osteoblasts were plated as above and subsequently transfected with a scrambled or TIEG1 specific siRNA, or infected with a control or TIEG1 adenovirus, for 24 hours respectively. Cells were then exposed to vehicle, TGFβ1 or BMP2 for 2 hours prior to analyzing Runx2 expression levels via real-time PCR.

### Expression Vectors and Reporter Constructs

Flag-tagged full length TIEG1 (1–480 aa) or truncated TIEG1 (1–370 aa) expression constructs were developed in our laboratory and cloned into the pcDNA4/TO expression vector (Invitrogen). The TIEG1 1–370 construct represents deletion of the DNA binding domain. The SIAH1 expression construct was also developed in our laboratory and cloned into pcDNA4/TO as previously described [46]. The flag-tagged TIEG1-NxN expression construct was kindly provided by Dr. Colin House and was developed as previously described [47]. The TIEG1-NxN construct contains two point mutations (V205B and P207B) which block SIAH1 interaction and result in accumulation of TIEG1 protein levels. The Runx2 promoter constructs and expression constructs were provided by Dr. Gary Stein's laboratory with the exception of the −600 to −101 bp construct which was cloned in our laboratory. All promoter constructs were ligated into the pGL3-basic luciferase vector (Promega, Madison, WI). The transcription factor binding site search using the −600 bp fragment of the Runx2 promoter as a template was performed using the Genomatix software suite (Munich, Germany). The p6OSE2-Luc reporter construct was originally developed by Dr. Patricia Ducy [48].

### Transient Transfection and Luciferase Assays

U2OS cells were plated in 12 well plates at a density of approximately 50%. The following day, cells were transfected in triplicate with 250 ng of either full length (1–480 aa) or truncated (1–370 aa) TIEG1 expression constructs and 250 ng of indicated reporter constructs using Fugene 6 (Roche, Indianapolis, IN) as described by the manufacturer. Following incubation at 37°C for 24 hours, cells were washed twice with PBS, lysed in 1X Passive Lysis Buffer (Promega) and equal quantities of protein extract were assayed for luciferase activity using Luciferase Assay Reagent (Promega).

### Western Blotting

To determine TIEG1 and TIEG1-NxN protein levels following co-transfection with SIAH1 in U2OS cells, whole cell extracts were prepared. Equal amounts of extract were separated using SDS PAGE, transferred to PVDF and blocked overnight in 5% milk in 1X TBST. Membranes were probed with a monoclonal Flag antibody (M2, Sigma, St. Louis, MO) and TIEG1 protein levels were visualized using enhanced chemiluminescence (Amersham Biosciences, Piscataway, NJ).

### Chromatin Immunoprecipitation (ChIP) Assays

U2OS cells were plated at a density of approximately 50% in 100 mm tissue culture plates and transfected in triplicate with 5 µg of both the indicated reporter and expression constructs as specified above. Following incubation for 24 hours, ChIP assays were performed as previously described [2]. Immunoprecipitations were carried out using 0.5 µg of a M2-Flag specific monoclonal antibody (Sigma). Inputs were generated as above excluding the antibody immunoprecipitation. Semi-quantitative PCR and quantitative Real-Time PCR were conducted in triplicate on all samples and a representative data set is shown. Primers used in the PCR reactions were designed to amplify the Runx2 promoter and are listed in Table 1. Quantitative PCR values were calculated based on the threshold cycle (C_t_) and were normalized to input controls.

### Co-Immunoprecipitation Assays

U2OS cells were plated at a density of approximately 50% in 100 mm tissue culture plates and transfected as indicated with 5 µg of TIEG1 or Runx2 expression constructs. Following 24 hours of incubation, cells were washed twice with PBS and lysed in RIPA buffer. Equal amounts of cell lysates were immunoprecipitated at 4°C overnight using 1 µg of either rabbit IgG, a Runx2 M-70 specific antibody (Santa Cruz) or a TIEG1 specific polyclonal antibody (#992-generated in our laboratory). Protein complexes were purified using protein G beads. Complexes were separated by SDS-PAGE, transferred to PVDF and blocked in 5% milk overnight. Western blotting was performed using either a TIEG1 specific polyclonal antibody (#992) or a Runx2 M-70 specific antibody (Santa Cruz).

### Differentiation of Calvarial Osteoblasts

Wildtype and TIEG1 KO calvarial osteoblasts were plated in 6-well tissue culture plates and allowed to proliferate to confluence. Once confluent, cells were cultured in osteoblastic differentiation medium containing β-glycerophosphate and ascorbic acid in the presence of either a control or Runx2 adenovirus as described above. Differentiation medium was replaced every three days. Following 21 days of differentiation, cells were stained for calcified bone nodules using Alizarin Red (Sigma-Aldrich). Briefly, cells were washed in 1X PBS and fixed in 10% neutral buffered formalin overnight at room temperature. Cells were washed twice with PBS and stained with 2% Alizarin Red (v/v) ph 4.2 for 10 minutes. Cells were washed extensively with distilled water and subsequently scanned.
